# *Acomys cahirinus seurati* as a Potential Reservoir Host of *Leishmania major*

**DOI:** 10.3390/pathogens15030268

**Published:** 2026-03-03

**Authors:** Sergei Karlin, Barbora Bečvářová, Kamal Eddine Benallal, Tomáš Bečvář, Ghania Mezai, Mounir Zaabar, Mohamed Mefissel, Petr Volf, Jovana Sádlová

**Affiliations:** 1 Department of Parasitology, Faculty of Science, Charles University, Vinicna 7, 128 44 Prague, Czech Republic; karlins@natur.cuni.cz (S.K.); barbora.vojtkova@natur.cuni.cz (B.B.); kbenallal@pasteur.dz (K.E.B.); tomas.becvar@natur.cuni.cz (T.B.); volf@cesnet.cz (P.V.); 2 Laboratory of Arboviruses and Emergent Viruses, Institut Pasteur of Algiers, Algiers 16047, Algeria; 3 Laboratory of Eco Epidemiology of Parasitic Diseases and Population Genetics, Institut Pasteur of Algiers, Algiers 16047, Algeria; zemaghania@gmail.com (G.M.); zaabarmounir23@gmail.com (M.Z.); 4 Public Establishment of Nearby Health, Ibn-Sina, Illizi 33000, Algeria; sassi33dz@gmail.com

**Keywords:** *Leishmania major*, *Acomys cahirinus*, *Phlebotomus papatasi*, reservoir host, cutaneous leishmaniasis, sand fly, xenodiagnoses

## Abstract

Cutaneous leishmaniasis (CL) caused by *Leishmania major* is a zoonotic disease transmitted by phlebotomine sand flies. Identification of reservoir hosts is critical for understanding transmission and guiding control. While *Psammomys obesus* and *Meriones shawi* are recognized reservoirs in North Africa, the role of other rodents remains unclear. We experimentally assessed the reservoir potential of *Acomys cahirinus seurati* from southeastern Algeria. Animals were intradermally infected, and clinical signs and infectiousness to sand flies were monitored. Parasite persistence in skin and visceral tissues was measured by quantitative PCR. All animals developed localized, self-limiting lesions. Seven of sixteen individuals transmitted parasites to *Phlebotomus papatasi* between 10 and 25 weeks post-infection. Parasites were mostly restricted to the inoculation site, with limited dissemination to contralateral pinnae and hindpaws, and rare presence in spleen or liver. Notably, some animals were infectious without visible lesions, indicating that ulceration is not required for transmission. These findings show that *A. cahirinus seurati* can maintain *L. major* infection for up to 6 months and transmit parasites to sand flies, fulfilling key criteria of a reservoir host. Thus, this species may contribute to CL transmission in endemic foci lacking classical reservoirs, emphasizing the need to consider alternative rodent hosts in surveillance and control programs in North Africa.

## 1. Introduction

Cutaneous leishmaniasis (CL) is a zoonotic vector-borne disease caused by parasitic protozoans of the genus *Leishmania* Ross, 1903 (Kinetoplastida: Trypanosomatidae) and typically manifests as self-limiting cutaneous lesions. The parasites exhibit a digenetic life cycle, alternating between vertebrate hosts and blood-feeding female phlebotomine sand flies (Diptera: Psychodidae). CL is endemic in 89 countries worldwide, with the Eastern Mediterranean region and Algeria representing a major eco-epidemiological hotspot that accounts for approximately 79% of all newly reported cases [1]. Although CL has been present in Algeria for decades, its incidence has increased substantially in recent years, with recurrent local outbreaks and thousands of new human cases reported annually [2]. *Leishmania major* Yakimoff and Schokhor, 1914, is the dominant causative agent, whereas *Leishmania tropica* Wright, 1903, and *Leishmania infantum* Nicolle, 1908, occur less frequently and exhibit distinct eco-epidemiological patterns [3,4].

In the Maghreb region, *L. major* is primarily transmitted by *Phlebotomus papatasi* (Scopoli, 1786) and maintained in gerbil reservoir hosts, mainly *Psammomys obesus* Cretzschmar, 1828, and *Meriones shawi* (Duvernoy, 1842) [5]. Their reservoir competence is supported by both field and laboratory studies demonstrating high infection prevalence and prolonged infectiousness to sand fly vectors [6,7,8]. However, susceptibility to *L. major* is not restricted to gerbils, as shown by the involvement of *Microtus guentheri* (Danford & Alston, 1880) in Israel [9]. Notably, several endemic CL foci in Algeria lack any confirmed reservoir species, emphasizing the need to investigate alternative rodent hosts among the diverse rodent fauna of the country [10]. Recently, *Gerbillus amoenus* (de Winton, 1902) was proposed as a potential reservoir in Illizi, an area of sporadic CL outbreaks in the central Sahara [11]. The present study extends this work by evaluating the susceptibility of *Acomys cahirinus seurati* (Desmarest, 1819).

The genus *Acomys* I. Geoffroy, 1838, is widely distributed across Africa and the Middle East, comprising small, arid-adapted rodents commonly known as spiny mice. Taxonomic relationships within the genus remain unresolved [12]. Multilocus phylogenetic analyses identify the *A. cahirinus* group as one of five major lineages and the most genetically diverse, encompassing multiple clades awaiting formal taxonomic revision [13]. Although the Algerian form has been assigned species status by some authors (*Acomys seurati* Heim de Balsac, 1936) [10], its taxonomic status remains uncertain; therefore, the designation *A. cahirinus seurati* is adopted here, according to [12].

This study aimed to assess whether *A. cahirinus seurati* can sustain *L. major* infection and transmit the parasite to sand fly vectors, thereby fulfilling the criteria of a potential reservoir host in Algeria.

## 2. Materials and Methods

### 2.1. Rodents, Parasites and Sand Flies

A breeding colony of *A. cahirinus seurati* was established at the animal facility of the Pasteur Institute of Algiers using animals originating from Illizi, a province in the southeastern part of the central Sahara, Algeria (26°30′18″ N, 8°28′56″ E; 567 m Above See Level). A detailed description of the locality is provided elsewhere [11]. In 2022, experimental infections were performed on six animals in Algeria. Subsequently, four males and four females were transported to Czechia to establish a breeding colony at the animal facility of the Faculty of Science, Charles University, Prague.

Animals were housed in family groups in glass cages (40 × 60 × 55 cm) or T4 breeding containers (Velaz, Prague, Czech Republic) with bedding (Hapy Horse, SubliCZ.cz, Sojovice, Czech Republic), nesting material (Wood wool, Ratiboř, Czech Republic), and hay. Rodents were provided with a standard rodent diet (Krysík, SubliCZ.cz, Sojovice, Czech Republic), supplemented occasionally with fresh organic vegetables and *Zophobas* Lacordaire, 1859, larvae, and had access to water ad libitum. Environmental conditions were maintained at 22–25 °C, 40–60% relative humidity, and a 12 h light/12 h dark photoperiod.

A laboratory colony of *P. papatasi* originating from Turkey has been maintained at the Department of Parasitology, Charles University, Prague, under standard conditions (26 °C, 60–70% relative humidity, 14 h light/10 h dark photoperiod) with access to 50% sucrose, as previously described [14].

The human isolate *L. major* MHOM/DZ/09/LIPA100/MON-25 from the M’Sila region, Algeria, was used. Promastigotes were cultured in M199 medium (Sigma-Aldrich, Merck, Darmstadt, Germany) supplemented with 10% heat-inactivated fetal bovine serum (FBS, Sigma-Aldrich, Merck, Darmstadt, Germany), 1% BME vitamins, 2% sterile human urine, and 250 µg/mL amikacin (Medochemie Bohemia, Prague, Czech Republic).

### 2.2. Experimental Infections of Sand Flies

*Phlebotomus papatasi* females were experimentally infected with *L. major* as described previously [15]. Briefly, log-phase promastigotes were resuspended in heat-inactivated defibrinated ram blood at a concentration of 5 × 10^6^ parasites/mL. Sand flies were allowed to feed through a chick-skin membrane, and fully engorged females were maintained under standard insectary conditions. For rodent infections, sand fly females were dissected 8–10 days post-blood meal, when mature infections with accumulated metacyclic forms were present in the thoracic midguts.

### 2.3. Experimental Infections of Rodents

Experimental infections of rodents were first carried out in Algeria, as a preliminary experiment on a small number of individuals (6 females) infected with a higher dose (1 × 10^6^) of culture-derived promastigotes.

After the establishment of the colony in Prague, the offspring of the captured animals were tested with a natural infectious dose of promastigotes derived from the guts of experimentally infected sand flies in three independent repetitions: five males (A1–A5) in experiment A, three females and three males (B1–B6) in experiment B, and five males (C1–C5) in experiment C. Rodents aged 6–10 weeks were anesthetized using a ketamine (66 mg/kg) (Narkamon, Bioveta, Ivanovice na Hané, Czech Republic) and xylazine (26 mg/kg) (Xylapan, Vétoquinol, Nymburk, Czech Republic) mixture. Parasites isolated from thoracic midguts of infected sand flies were pooled in sterile saline and counted using a Bürker chamber. Salivary glands (SG) of *P. papatasi* females were pooled (10 glands per 10 µL saline), stored at −20 °C, and subjected to three freeze–thaw cycles in liquid nitrogen prior to use. The resulting SG lysate was mixed with the parasite suspension and intradermally injected into the left ear pinna in a total volume of 5.5 µL. Each animal received 2–5 × 10^4^ parasites and 0.5 SG.

The proportion of metacyclic promastigotes in the inoculum was determined from methanol-fixed, Giemsa-stained smears (Sigma-Aldrich, Merck, Darmstadt, Germany). Parasites were examined by light microscopy, photographed using an Olympus DP70 camera (Olympus Czech Group, Prague, Czech Republic), and measured with ImageJ software (https://imagej.net/software/imagej/) [15]. Promastigotes were classified as metacyclic when the flagellum length was at least twice the body length.

Animals were monitored weekly by visual inspection for clinical signs of infection and subjected to xenodiagnosis at 5-week intervals for up to 25 weeks post-infection (Prague experiments).

### 2.4. Xenodiagnosis

Rodents were anesthetized with ketamine/xylazine, and 30–40 female *P. papatasi* were allowed to feed on the inoculated pinnae using small plastic tubes covered with fine nylon mesh. On days 7–8 post-blood meal, sand flies were dissected, and their guts were examined by light microscopy. Infection intensity and localization were assessed as described previously [16].

### 2.5. Tissue Sampling and Parasite Detection

Rodents were euthanized by overdose with anesthetic. The following tissues were collected: whole inoculated and contralateral pinnae, ear-draining lymph nodes, spleen, liver (approximately one quarter), forepaws, hindpaws, and tail skin. Samples were stored at −20 °C until processing.

Total DNA was extracted using a tissue DNA isolation kit (Roche Diagnostics, Mannheim, Germany) according to the manufacturer’s instructions. Parasite loads were quantified by quantitative PCR (qPCR) using a LightCycler^®^ 480 system and SYBR Green chemistry (LightCycler^®^ 480 SYBR^®^, Green I Master, Mannheim, Germany). Primers targeting a 116 bp fragment of *Leishmania* kinetoplast minicircle DNA were used (forward primer (13A): 5′-GTGGGGGAGGGGCGTTCT-3′ and reverse primer (13B): 5′-ATTTTACACCAACCCCCAGTT-3′). PCR conditions consisted of an initial denaturation at 95 °C for 5 min, followed by 40 cycles of 95 °C for 10 s, 61 °C for 20 s, and 72 °C for 10 s. Standard curves were generated using 10-fold serial dilutions of *L. major* DNA corresponding to 5 × 10^5^ to 5 × 10^−1^ parasites per reaction. Quantification was performed by interpolation from the standard curve, and melting curve analysis (72–95 °C) was used to verify amplification specificity.

## 3. Results

### 3.1. Preliminary Experiment

Six animals were infected in Algeria with 1 × 10^6^ culture-derived *L. major* promastigotes and monitored for 15 weeks. Nodules, representing the first external signs of infection, appeared 6–7 weeks post-infection (p.i.) In two animals, nodules resolved within two weeks, and no further clinical signs developed. In contrast, four animals progressed to ulcerative lesions; lesion healing occurred in one individual by 14 weeks p.i., whereas lesions persisted in the remaining three animals until the end of the experiment. Post-mortem PCR analysis detected *Leishmania* DNA in all inoculated pinnae. Parasite DNA was additionally detected once in the contralateral pinna and in two cases each in the liver and spleen.

### 3.2. Course of Infection and Xenodiagnosis

After establishment of the colony in Prague, a total of 16 *A. cahirinus seurati* were infected in three independent repetitions (A, B, C) with (2–5) × 10^4^ sand fly–derived promastigotes plus 0.5 SG equivalents per animal. The proportion of metacyclic forms in the inocula was 33%, 18%, and 17% in experiments A, B, and C, respectively.

**Figure 1:**
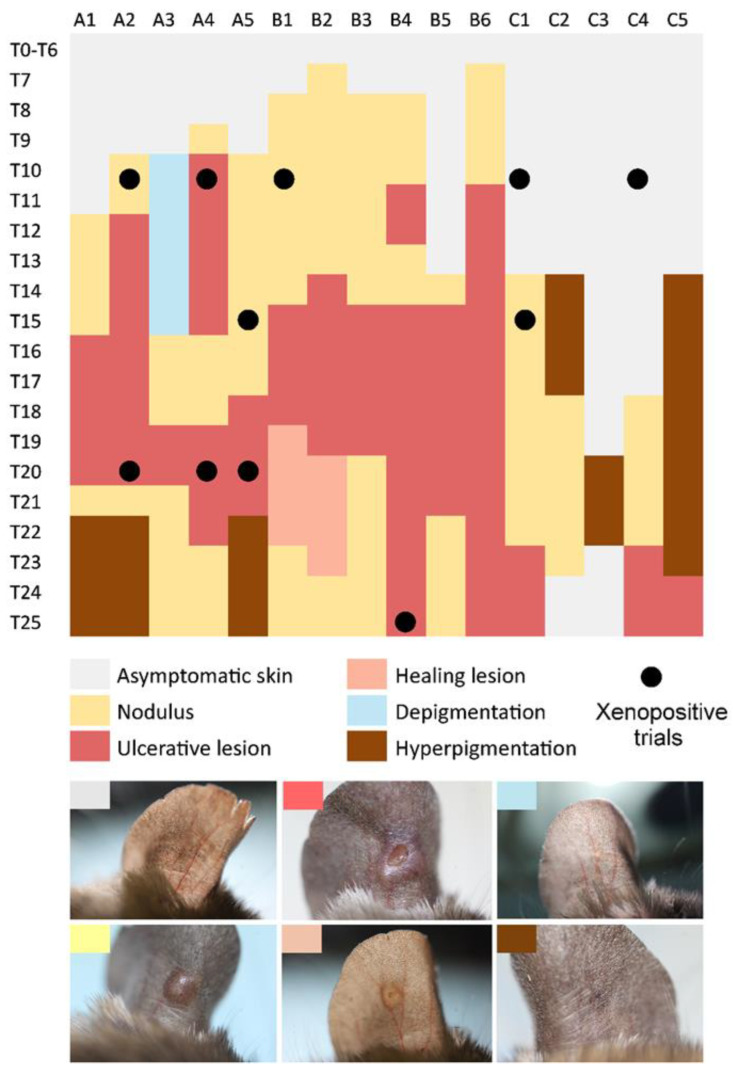
Skin manifestation of *L. major* in *Acomys cahirinus seurati*. Xenodiagnosis with a positive result is marked with a black circle. A1–C5, animal ID; T1–T25, week post-infection.

All animals developed cutaneous manifestations at the inoculation site during the 25-week observation period (Figure 1). In experiments A and B, the first visible signs appeared 7–10 weeks p.i., whereas in experiment C, the asymptomatic period lasted at least 14 weeks. The earliest clinical manifestation was typically a nodule, which progressed to an ulcerative lesion within 1–9 weeks. Lesions resolved within 2–9 weeks in most animals; however, in five individuals, lesions persisted until the end of the experiment (25 weeks p.i.). Given the overall self-limiting course of infection, lesion healing in these animals would likely have occurred with longer follow-up. Post-healing hyperpigmentation was observed in three animals, whereas in three others, hyperpigmentation preceded lesion development, and in one animal it represented the only skin manifestation. Transient depigmentation was recorded in a single individual for six weeks prior to nodule formation (Figure 1).

Xenodiagnosis was performed at five-week intervals, during which a total of 1598 female *P. papatasi* were allowed to feed on anesthetized rodents. Overall, 44% of animals (7/16) were infectious to sand flies, with four individuals transmitting parasites repeatedly. Infectiousness was most frequently detected at 10 weeks p.i., although positive xenodiagnostic results were also obtained at 15, 20, and 25 weeks p.i. (Figure 1; Table S1).

### 3.3. Post-Mortem Analysis

**Figure 2:**
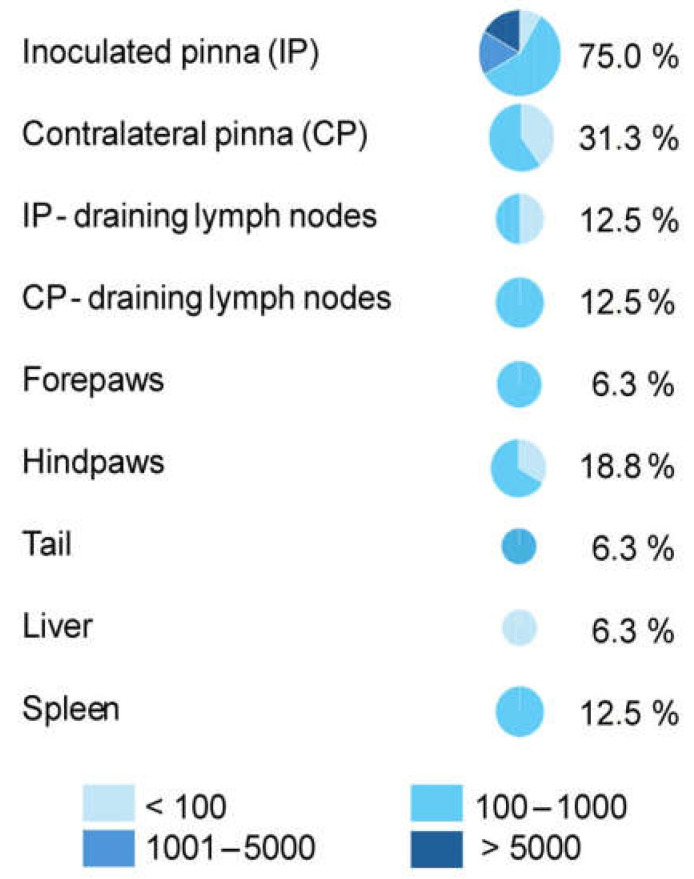
*Leishmania major* in tissues of *Acomys cahirinus seurati*: percentage of infected animals and parasite load measured by qPCR.

Post-mortem analyses revealed the presence of *Leishmania* DNA in all animals except individual A5, in which lesion healing was nearly complete by 21 weeks p.i., four weeks prior to the end of the experiment. Parasites were most frequently detected in the inoculated pinna (75% of animals; Figure 2). However, dissemination to other peripheral tissues was also observed, particularly to the contralateral pinna (31%) and hindpaws (19%). Visceral dissemination was rare, with parasite DNA detected in the spleen of 12% and in the liver of 6% of animals. Most tissues harbored low parasite loads (100–1000 parasites), whereas moderate (1000–5000) and high (>5000) parasite burdens were detected exclusively in the inoculated pinnae (Figure 2).

## 4. Discussion

This study provides novel experimental evidence supporting the inclusion of *A. cahirinus* among potential rodent reservoir hosts of *L. major* in North Africa. Identification of reservoir hosts is a critical prerequisite for the effective control of zoonotic diseases; however, it requires long-term, integrative field and laboratory investigations.

Recent parasitological surveys have revealed the presence of *L. major* DNA in a broad range of rodent species across North Africa. In addition to the well-established reservoir hosts *P. obesus* and *M. shawi*, natural infections have been documented in several other rodents, including *Meriones libycus* Lichtenstein, 1823, in Libya [17,18]; *Ctenodactylus gundi* (Rothman, 1776)*, Jaculus jaculus* (Linnaeus, 1758) and *Jaculus hirtipes* (Lichtenstein, 1823) in Tunisia [19,20]; and *Meriones crassus* Sundevall, 1842, *Meriones sacramenti* Thomas, 1922, *Rattus rattus* (Linnaeus, 1758), *Rattus norvegicus* (Berkenhout, 1769), *Gerbillus pyramidum* I. Geoffroy, 1803, and *Gerbillus andersoni* de Winton, 1902, in Egypt [21,22,23]. Beyond rodents, *L. major* DNA has also been detected in insectivores and carnivores, including hedgehogs (*Atelerix algirus* Lereboullet, 1842, *Paraechinus aethiopicus* (Ehrenberg, 1832)) and the weasel *Mustela nivalis* Linnaeus, 1766 [24,25].

Several *Leishmania* species have previously been reported in rodents of the genus *Acomys*. Early evidence originates from Sudan, where inoculation of spleen material from *Acomys albigena* (Cretzschmar, 1828) resulted in infection of laboratory hamsters in a focus endemic for *Leishmania donovani* (Leishman & Donovan, 1903) [26]. More recently, *L. tropica* was detected molecularly in *Acomys* spp. in southern Ethiopia [27], and *L. major* DNA was identified in *Acomys dimidiatus* (Cretzschmar, 1826) in Iran [28]. However, detection of parasite DNA alone does not demonstrate reservoir competence.

To qualify as a reservoir host, a species must support long-term parasite persistence—ideally across at least one non-transmission season—and allow parasite localization compatible with sand fly infection [29]. Otherwise, infected animals may act only as parasite sinks, contributing little or nothing to onward transmission [30]. For this reason, laboratory-based experimental infections combined with xenodiagnosis are essential complements to field studies.

Our experimental data demonstrate that *A. cahirinus seurati* can remain infectious to *P. papatasi* for up to 25 weeks post-infection, with parasites predominantly persisting in the skin at the site of inoculation. Visceral dissemination to the spleen and liver was rare, which is consistent with observations in most rodent hosts of *L. major* [8,31,32]. Cutaneous lesions followed a self-limiting course and healed without extensive tissue destruction, an outcome consistent with the exceptional regenerative capacity of *Acomys* skin [33].

Importantly, the presence of ulcerative skin lesions is not a prerequisite for infectiousness to sand fly vectors. Previous studies have shown that asymptomatic *M. shawi* individuals were as infectious to *P. papatasi* as animals with active lesions [8]. Similarly, experimental infections of other African rodent species proposed as potential *L. major* reservoirs, such as *Mastomys* Thomas, 1915, and *Arvicanthis* Lesson, 1842, resulted only in mild skin alterations, including hyperpigmentation, without ulcer formation [32].

In the present study, *Acomys* individuals were infectious to sand flies from week 10 to week 25 post-infection; however, the overall proportion of infected *P. papatasi* females was low (0.8%). This finding aligns with previous observations indicating that even in hosts with substantial skin parasite loads, only a fraction of feeding sand flies acquire infection, likely due to the heterogeneous and patchy distribution of parasites in the skin [34,35]. In *M. shawi*, infection rates reached 11%, with a strong dependence on the precise feeding site: 37.9% of sand flies feeding at lesion margins became infected, compared with only 1.4% feeding on intact skin [35].

The present study was conducted under controlled experimental conditions using a single, well-characterized *L. major* strain and the primary vector species *P. papatasi*, which is considered the dominant and epidemiologically most relevant vector of *L. major* in North Africa. While this design ensured biological relevance and experimental reproducibility, future studies may examine whether reservoir competence of *Acomys* spp. varies among different parasite genotypes circulating across endemic regions. In addition, the study focused on a single population of *A. cahirinus seurati* originating from southeastern Algeria. Given the unresolved taxonomy and substantial genetic diversity within the genus *Acomys*, reservoir competence may differ among geographically or genetically distinct populations, which warrants further investigation.

## 5. Conclusions

In conclusion, this study provides the first experimental evidence that *A. cahirinus seurati* fulfills key criteria of a potential reservoir host for *L. major*, including sustained parasite persistence in the skin and the ability to infect *P. papatasi* over an extended period up to 6 months. Together with previous findings on *G. amoenus*, our results suggest that alternative rodent hosts may play an important role in maintaining *L. major* transmission in endemic foci where the classical reservoirs *P. obesus* and *M. shawi* are absent. This scenario is particularly relevant for sparsely populated Saharan regions such as Illizi in southeastern Algeria, where local ecological conditions favor distinct host assemblages. Recognition of such non-classical reservoir hosts is essential for improving risk assessment and designing targeted surveillance and control strategies for cutaneous leishmaniasis in North Africa.

## Data Availability

Data are contained within the article or Supplementary Materials.

## Supplementary Materials

The following supporting information can be downloaded at: https://www.mdpi.com/article/10.3390/pathogens15030268/s1, Table S1: Results of xenodiagnostic experiments performed with *Phlebotomus papatasi* on *Acomys cahirinus seurati* infected with *Leishmania major* in Prague.

## Abbreviations

The following abbreviations are used in this manuscript:

CL	Cutaneous leishmaniasis
qPCR	Quantitative Polymerase Chain Reaction
FBS	Fetal bovine serum
p.i.	Post-infection
IP	Inoculated pinna
CP	Contralateral pinna
SG	Salivary glands
