# Supplementary material for: Acomys cahirinus seurati as a Potential Reservoir Host of Leishmania major
Source: Pathogens. 2026 Mar 3;15(3):268. doi: 10.3390/pathogens15030268 (PMC13029579; doi:10.3390/pathogens15030268)
Supplement: Supplementary file 1 [file pathogens-15-00268-s001.zip › pathogens-4165886-Supplementary Material.pdf]

Table S1 Results of xenodiagnostic experiments performed with *Phlebotomus papatasi* on *Acomys cahirinus seurati* infected with *Leishmania major*.

Week p.i.	*Acomys* No.	Dissected females	Positive females	*Acomys* No.	Dissected females	Positive females	*Acomys* No.	Dissected females	Positive females
5	A1	15	0	B1	20	0	C1	11	0
10		20	0		20	1		27	1
15		19	0		21	0		8	1
20		22	0		22	0		24	0
25		23	0		20	0		32	0
5	A2	17	0	B2	21	0	C2	11	0
10		19	1		18	0		21	0
15		21	0		13	0		20	0
20		17	2		20	0		13	0
25		24	0		17	0		23	0
5	A3	19	0	B3	20	0	C3	20	0
10		22	0		16	0		27	0
15		25	0		19	0		15	0
20		20	0		21	0		13	0
25		24	0		29	0		30	0
5	A4	17	0	B4	20	0	C4	15	0
10		22	1		11	0		17	1
15		16	0		19	0		11	0
20		23	1		24	0		19	0
25		18	0		26	1		36	0
5	A5	21	0	B5	20	0	C5	15	0
10		22	0		14	0		19	0
15		13	1		18	0		28	0
20		13	2		22	0		17	0
25		24	0		30	0		31	0
5				B6	21	0			

10			12	0		
15			22	0		
20			25	0		
25			18	0		
∑	496	8	599	2	503	3
